# Associations Between 25-Hydroxyvitamin D and Total and γ' Fibrinogen and Plasma Clot Properties and Gene Interactions in a Group of Healthy Black South African Women

**DOI:** 10.3389/fcvm.2022.868542

**Published:** 2022-07-12

**Authors:** Petro H. Rautenbach, Cornelie Nienaber-Rousseau, Zelda de Lange-Loots, Iolanthé M. Kruger, Marlien Pieters

**Affiliations:** ^1^Centre of Excellence for Nutrition, North-West University, Potchefstroom, South Africa; ^2^Medical Research Council Unit for Hypertension and Cardiovascular Disease, North-West University, Potchefstroom, South Africa; ^3^Africa Unit for Transdisciplinary Health Research (AUTHeR), North-West University, Potchefstroom, South Africa

**Keywords:** cholecalciferol, ergocalciferol, fiber density, fibrinolysis, lag time, lateral aggregation, nutrigenetics, turbidity

## Abstract

The role of 25-hydroxyvitamin D [25(OH)D] in reducing the risk of cardiovascular disease (CVD) has been recognized, but the mechanisms involved are unclear. Researchers have discovered a link between vitamin D and fibrinogen. Until now, data on the relationship between vitamin D and the γ' splice variant of fibrinogen and fibrin clot characteristics remain unexplored. In this study, 25(OH)D, total and γ' fibrinogen, as well as turbidimetrically determined plasma clot properties, were quantified, and fibrinogen and *FXIII* SNPs were genotyped in 660 Black, apparently healthy South African women. Alarmingly, 16 and 45% of the women presented with deficient and insufficient 25(OH)D, respectively. Total fibrinogen and maximum absorbance (as a measure of clot density) correlated inversely, whereas γ' fibrinogen correlated positively with 25(OH)D. γ' fibrinogen increased whereas maximum absorbance decreased over the deficient, insufficient, and sufficient 25(OH)D categories before and after adjustment for confounders. 25(OH)D modulated the association of the SNPs regarding fibrinogen concentration and clot structure/properties, but did not stand after correction for false discovery rate. Because only weak relationships were detected, the clinical significance of the findings are questionable and remain to be determined. However, we recommend vitamin D fortification and supplementation to reduce the high prevalence of this micronutrient deficiency and possibly to improve fibrinogen and plasma clot structure if the relationships are indeed clinically significant. There is a need for large cohort studies to demonstrate the relationship between vitamin D and cardiovascular and inflammatory risk factors as well as to uncover the molecular mechanisms responsible.

## Introduction

Although cardiovascular (CVD) and other non-communicable diseases are major causes of mortality (1, 2), these conditions also increase the risk of dying of communicable diseases such as coronavirus disease (COVID-19) (3–5). A link between deficiency of vitamin D—a group of fat-soluble secosteroids with the most significant being vitamin D_3_ (cholecalciferol) and D_2_ (ergocalciferol), well-known for their role in bone health—and CVD, has been proposed (6–8). However, the underlying mechanisms remain to be clarified. One route that has been recognized is that vitamin D affords protection against circulatory diseases by reducing inflammation (9–12). Less-studied pathways include improving the blood lipid profile (13), downregulation of the renin-angiotensin system improving blood pressure (14, 15), enhancement of insulin secretion and insulin sensitivity (16, 17), protection against uncontrolled angiogenesis (18) and influencing blood coagulation (19, 20).

As vitamin D can be acquired through diet and cholecalciferol synthesized in a chemical reaction when the skin is exposed to sunlight, circulating concentrations of 25-hydroxyvitamin D-−25(OH)D, the sum of vitamin D_2_ and D_3_ and the precursor of the active form 1,25-dihydroxyvitamin D_3_ (1,25(OH)D)—is the hallmark of assessing vitamin D status. The relationships between 25(OH)D concentrations and inflammatory markers, including the glycoprotein fibrinogen, have been investigated in numerous studies, with most sampling small or specific patient groups (19–24). Data for the association between vitamin D and fibrinogen in generally healthy populations is sparse, and evidence from continental Africans, with darker skins that hinder vitamin D production, is limited with some reports on African Americans (12, 25). Whereas some researchers have detected an inverse relationship between vitamin D and fibrinogen (19, 20, 24, 26, 27), information regarding the fibrinogen isoform that stems from a splice variant of the γ-chain messenger RNA resulting from an alternative polyadenylation signal in intron 9, i.e., fibrinogen γ', and clot properties is lacking. Investigating the relationship between fibrinogen and vitamin D is crucial because fibrinogen is the dominant coagulation protein in blood by mass and the precursor of fibrin, the main constituent of blood clots (28–30). Studying fibrinogen γ' is vital because of its influence on clot properties (31–33). Factor XIII (FXIII) is another blood coagulation factor that influences fibrin clot structure and functions by cross-linking fibrin fibers to prevent premature lysis of the clot to allow wound healing (34, 35). Furthermore, total and γ' fibrinogen concentrations, as well as clot structure and characteristics, may be influenced by single nucleotide polymorphisms (SNPs) within the fibrinogen α, β and γ chain genes (*FGA, FGB* and *FGG*) as well as those of *FXIII* (36). Therefore, fibrinogen and *FXIII* SNPs might interact with 25(OH)D to modulate total or γ' fibrinogen concentration as well as the kinetics of clot formation, structural clot properties and fibrinolysis and warrant consideration.

Thus, we first investigated the associations of 25(OH)D determined in a group of ostensibly healthy Black women with total and γ' fibrinogen and fibrin clot properties. Second, we determined how these associations are influenced by specific genetic variations within the *FGA, FGB, FGG* and *FXIII* genes. To provide possible explanations for the observed relationships we considered the inflammatory marker C-reactive protein (CRP) and glycated hemoglobin A1c (HbA1c) which is a long-term indicator of glucose control. By controlling for CRP and HbA1c we explored whether the underlying mechanisms were related to inflammation or to vitamin D's effect on glucose control (16, 17). This research is pressing because unraveling probable mechanisms for the observed relationship between vitamin D and CVD could inform the design of targeted approaches to prevent CVD in those most vulnerable.

## Materials and Methods

### Research Design and Ethical Considerations

The Prospective Urban and Rural Epidemiology (PURE) study investigated the development of chronic lifestyle diseases, especially CVDs, by stratifying populations at different levels of urbanization (37). For this cross-sectional investigation, we used a subset of 660 randomly selected women derived from the 1,263 healthy women older than 30 years who participated in the North West site of the South African arm of the PURE study at baseline. Goodwill permission was granted by mayors, community leaders and tribal chiefs before researchers accessed the communities included for recruitment. Eligible individuals were well-informed regarding all aspects of the research and, after ample time for reflection, were asked to sign an informed consent form indicating their agreement to join in the study. Final enrolment and measurements for baseline were scheduled between August and November 2005, which is late winter to late spring in the Southern Hemisphere. Due to monetary constraints vitamin D was not measured in the follow-up blood samples. Persons with elevated tympanic temperatures (above 38°C) were excluded. Additional exclusion criteria were using chronic medication (apart from blood pressure medicine), having any known lifestyle disease, being pregnant or lactating, or having a known infection, such as tuberculosis-causing agents and/or human immunodeficiency virus (HIV). However, participants diagnosed with HIV during recruitment were included in the study and, because total fibrinogen, %γ' fibrinogen, lag time, slope, maximum absorbance, clot lysis time (CLT) and 25(OH)D did not differ between infected or non-infected, were also included in the statistical analysis.

Ethical approval of the study protocol was obtained for the larger study as well as this nested study from the Health Research Ethics Committee of the Faculty of Health Sciences, North-West University (NWU–HREC, ethics number: 04M10; NWU-00016-10-A1 and NWU-00034-17-A1-02), honoring the guidelines laid down in the Declaration of Helsinki of 1975 and revised in 2000.

### Blood Pressure Measurements

Blood pressure—systolic and diastolic—was measured twice using the OMRON HEM-757 automatic apparatus (Omron Healthcare, Kyoto, Japan) with the cuff on the left arm after 5 min of sitting in a calm environment. The second measurement was recorded.

### Anthropometric Measurements

While participants were lightly clothed and their arms hung freely at their sides, their body weights were recorded twice. Heights were measured twice using stadiometers with volunteers' heads in the Frankfort plane, bodies fully extended while inhaling. The means of the participants' weight and height measurements were used, to compute body mass index. Waist circumference was determined at the narrowest point between the 10^th^ rib and iliac crest. Hip circumference was assessed at the level where the protrusion of the buttocks was greatest.

### Blood Collection, Sampling, and Storage

Blood samples were collected between 07:00 and 11:00 from fasting participants. Sodium citrate-treated (3.8%) whole blood was centrifuged at 2,000 x g for 15 min to yield a buffy coat for DNA extraction and plasma samples to quantify coagulation markers. Ethylenediamine tetraacetic acid-treated whole blood was collected for HbA1c determination. Coagulated blood was collected to yield serum for lipids, CRP, and γ-glutamyl transferase (GGT) analysis. Aliquots were frozen on dry ice pellets, stored in the field at −18°C and then, after 2–4 days, at −80°C until analyzed.

### Biochemical Analysis

Lipograms [including total cholesterol (TC), high-density lipoprotein cholesterol (HDL-C) and triglycerides], high sensitivity CRP and GGT concentrations were assayed using a Sequential Multiple Analyzer (Konelab 20i, Thermo Fisher Scientific Oy, Vantaa, Finland). Low-density lipoprotein cholesterol (LDL-C) was calculated using the Friedewald formula for triglyceride concentrations below 400 mg/dL. HbA1 concentrations were determined using the D-10 Hemoglobin testing system (Bio-Rad Laboratories, Hercules, CA, USA). Serum total 25(OH)D concentrations were quantified using a Roche Elecsys 2010 COBAS system (functional sensitivity: 10.0 nmol/L; Roche Diagnostics, Indianapolis, IN, USA). Whole blood was used for a rapid first response HIV card test 1–2.0 (Transnational Technologies Inc. PMC Medical, Nani Daman, India), and the outcome was confirmed with a Pareeshak test (BHAT Bio-tech, Bangalore, India).

Total fibrinogen concentrations were established with the adapted Clauss method (Multifibrin U-test, BCS coagulation analyzer, Dade Behring, Deerfield, IL, USA). Fibrinogen γ' was quantified with an ELISA using a 2.G2.H9 mouse monoclonal coating antibody against the human γ' sequence (Santa Cruz Biotechnology, Santa Cruz, USA) for antigen capture and a goat polyclonal antibody against human fibrinogen (Antibody 7,539, Abcam for development, Cambridge, USA). Turbidimetric analysis (A405 nm) was used (38) with modified tissue factor and tissue-plasminogen activator (tPA) concentrations to attain plasma fibrinolytic potential reported as CLTs of around 60 to 100 min. Final concentrations were tissue factor (125x diluted – an estimated final concentration of 59 pM; Dade Innovin, Siemens Healthcare Diagnostics Inc., Marburg, Germany), CaCl_2_ (17 mmol/L), tPA (100 ng/mL; Actilyse, Boehringer, Ingelheim, Germany) and phospholipid vesicles (10 μmol/L; Rossix, Mölndal, Sweden). Kinetics of clot formation (with lag time presenting the time required for fibrin fibers to attain the appropriate length to allow lateral aggregation as well as activation of the coagulation cascade and slope representing the rate of lateral aggregation), and structural clot properties (maximum absorbance representing clot density) were computed from the turbidity curves. The coefficient of variation for all assays was <10%.

### Factors Pertaining to Lifestyle

Participants responded to various validated interviewer-administered questionnaires in a language of their choice to obtain demographic and lifestyle information.

### Genetic Analyses

For details on the genetic analyses, please refer to Cronjé, Nienaber-Rousseau (39). In short, genomic DNA was isolated from buffy coat using the FlexiGene™ kits (QIAGEN Inc., Valencia, CA, USA). SNPs selected from the literature [*FGB*-rs7439150, rs1800789 (1420G/A), rs1800791 (−854G/A), rs1800790 (−455G/A), rs1800788 (249C/T), rs1800787 (−148C/T), rs4220, rs4463047, *FGA*-rs6050, rs2070011 (2224G/A), and *FGG* rs2066865 and rs1049636 (9340T/C) as well as *FXIII* His95Arg A/G (rs6003) and Val34Leu, C/A (rs5985)], and two novel *FGB* SNPs in the promoter region (rs2227385 and rs2227388) identified *via* sequencing a subpopulation were genotyped. The Thermo Fischer Scientific® Taqman based assay, Illumina® VeraCode GoldenGate assay technology using a BeadXpress® platform and competitive allele-specific polymerase chain reactions (KASP) were used for genotyping.

### Statistical Analyses

Statistica® version 13.3 (TIBCO Software Inc., Tulsa, OK, USA) and R version 3.5.0 (40) were used for analysis. Normally distributed data are expressed as mean [95% confidence intervals (CI)], and not-normally distributed data as median (lower and upper quartiles). Differences between HIV and urbanization status in terms of 25(OH)D and coagulation factors were determined by Mann–Whitney U tests and among tobacco use subgroups, using Kruskal–Wallis analysis of variance (ANOVA). Spearman correlations were performed to test for statistical dependence between variables and identify confounders and/or co-variates for inclusion in subsequent analysis (age, LDL-C, body mass index (BMI), HbA1c, GGT, and CRP). Spearman partial correlations were conducted adjusting for these co-variates and additionally for fibrinogen when the outcome variables were clot properties to determine whether the relationship between vitamin D and clot properties was influenced by fibrinogen concentration. The following categories were created based on vitamin D status <50 nmol/L (deficient), 50–75 nmol/L (insufficient) and >75 nmol/L (sufficient) (41, 42). General linear models were used to determine differences in fibrinogen and clot properties among the vitamin D status categories.

To investigate whether vitamin D has interactive effects with the fibrinogen and *FXIII* gene polymorphisms in predicting total and %γ' fibrinogen concentration and clot properties, general linear models (adjusting for age, LDL-C, BMI, HbA1c, GGT, and CRP) with interactions were adopted. Since the frequency of carrier homozygote variant carrier status was low, we group the genotypes using a dominant genetic mode of action, that is, combining the heterozygotes with those that were homozygous for the variant alleles (0/1/1). Only interactions with a *p*-value < 0.05 are presented; however, for multiple testing and false discovery rates, the statistical threshold for significance was *p* < 0.005 [1/(16^*^3)(0.25)] when the false discovery rate was set at 25% (43), the number of SNPs investigated was 16, the vitamin D one, whereas total and γ' fibrinogen concentrations and fibrin clot properties were regarded as three due to co-linearity. To describe the interaction, Spearman partial correlations were used, adjusting for age, LDL-C, BMI, HbA1c, GGT, and CRP and additionally for fibrinogen, when the interaction was concerning clot properties.

## Results

### Characteristics of the Study Population in Terms of Vitamin D Status, Fibrinogen, and Clot Properties

In Table 1, we summarize the demographic and clinical characteristics of the women constituting our sample. The median 25(OH)D concentration was 69.5 nmol/L; 16% of the women presented with vitamin D deficiency, 45% with insufficiency and 39% with adequate status.

**Table 1:** Specific characteristics of a group (*n* = 660) of Black South African women.

**Characteristics**		**Median (interquartile range) or** ***n*****:** ***n*** **or** ***n*****, %**		
		**Whole group**	**Rural**	**Urban**
Age (yr)		54.0 (49.0–60.0)	47.0 (41.0–54.0)	48.0 (42.0–58.0)
Urbanization (Rural: Urban)		335:325	–	–
Blood pressure (mmHg)	SBP	136 (121–154)	124 (112–141)	133 (119–150)
	DBP	90 (81–99)	86.0 (77.0–95.0)	89.0 (80.0–99.0)
Waist circumference (cm)		82.0 (72.0–91.5)	78.8 (69.5–89.5)	82.8 (73.1–92.8)
Hip circumference (cm)		100 (91.0–111)	99.0 (89.6–111)	103 (91.8–114)
WHR		0.81 (0.76–0.86)	0.80 (0.76–0.84)	0.80 (0.76–0.85)
Weight (kg)		63.3 (52.9–77.1)	61.2 (51.1–1.61)	66.6 (54.7–81.8)
Height (cm)		157 (152–160)	1.57 (1.53–1.61)	1.56 (1.52–1.61)
BMI (kg/m^2^)		26.1 (22.0–31.3)	24.9 (20.7–30.6)	27.6 (22.5–32.7)
HIV status (Negative: Positive)		597:59	306:28	291:31
Biochemical markers	25(OH)D, (ng/ml; nmol/L)	27.8 (22.3–33.5); 69.5 (55.8–83.8)	28.7 (24.3–34.5); 71.6 (60.8–86.2)	26.9 (21.4–32.4); 67.3 (53.6–81.0)
	TC (mmol/L)	5.24 (4.41–6.29)	5.17 (4.33–6.21)	5.30 (4.48–6.33)
	LDL-C (mmol/L)	3.09 (2.32–3.97)	2.85 (2.2–3.76)	2.95 (2.24–3.77)
	HDL-C (mmol/L)	1.41 (1.09–1.82)	1.39 (1.09–1.84)	1.36 (1.03–1.78)
	Triglycerides (mmol/L)	1.27 (0.92–1.79)	1.09 (0.82–1.49)	1.18 (0.87–1.78)
	HbA1c (%)	5.70 (5.40–6.00)	5.60 (5.30–5.80)	5.60 (5.30–5.90)
	CRP (mg/L)	4.33 (1.50–12.2)	3.51 (1.04–9.33)	4.11 (1.45–12.0)
	GGT (U/L)	42.0 (29.0–64.5)	938 (690–1,157)	190 (49.0–373)
	Total fibrinogen (g/l)	3.30 (2.50–5.80)	3.20 (2.50–5.70)	2.90 (2.30–5.40)
	Fibrinogen γ' (%)	9.76 (6.75–14.5)	10.1 (6.35–14.9)	10.7 (7.86–15.5)
	Lag time (min)	6.60 (5.22–7.84)	5.83 (4.52–7.14)	7.17 (5.72–8.58)
	Slope (AU/s)	9.31 (6.76–12.3)	10.2 (7.64–13.3)	7.41 (5.57–10.3)
	Maximum absorbance (nm)	0.45 (0.35–0.54)	0.41 (0.32–0.50)	0.44 (0.35–0.54)
	CLT (min)	58.8 (52.6–65.9)	56.3 (51.3–62.9)	60.2 (54.7–66.8)

*Total sample was 660, but numbers are slightly different for several variables due to missing data*.

*AU, absorbance units; 25(OH)D, 25-hydroxyvitamin D; BMI, body mass index; CLT, clot lysis time; CRP, C-reactive protein; DBP, diastolic blood pressure; GGT, γ-glutamyl transferase; HbA1c, glycated hemoglobin A1c; HDL-C, high-density lipoprotein cholesterol; HIV, human immunodeficiency virus; LDL-C, low-density lipoprotein cholesterol; mmHg, millimeters of mercury; SBP, systolic blood pressure; TC, total cholesterol; WHR, waist-hip ratio*.

*Conversion: 1 nmol/L 25(OH)D = 0.4 ng/mL 25(OH)D = 0.0004 mg/L 25(OH)D*.

As can be seen from the table above, 25(OH)D differed between the rural and urban women, with the former presenting with higher concentrations. Total and %γ' fibrinogen, time to clot formation indicated by lag time and the rate of lateral aggregation as indicated by slope also differed. Total fibrinogen and slope were higher in the rural than urban participants, whereas %γ' fibrinogen and lag time were higher in the urban than rural living participants. Clot density as indicated by maximum absorption and CLT did not differ between the urbanization subgroups. No difference in 25(OH)D concentration, fibrinogen, % γ' fibrinogen, lag time, slope, maximum absorbance, or CLT was detected between HIV-seropositive and -negative women.

### Association of 25(OH)D Concentrations With Circulating Fibrinogen and Clot Properties

In Table 2, we present the correlations between 25(OH)D and fibrinogen as well as clot properties. Noteworthy observations were total fibrinogen correlating inversely, whereas γ' fibrinogen correlated positively with 25 (OH)D and a negative correlation between maximum absorbance and 25(OH)D. However, after adjusting for variables including CRP (r = 0.42; *p* < 0.001 with fibrinogen) as well as HbA1c (*r* = 0.20; *p* < 0.001 with fibrinogen), only the correlations between γ' fibrinogen and lag time remained and, after further adjustment with fibrinogen, only lag time remained worth mentioning.

**Table 2:** Correlations of 25(OH)D with total fibrinogen and γ' fibrinogen concentrations and plasma clot properties.

**Fibrinogen and clot properties**	**Model 1**	**Model 2**	**Model 3**
	**Spearman rho;** ***p*** **value**		
Total fibrinogen	−0.10; 0.01	−0.07; 0.09	–
% Fibrinogen γ'	0.11; 0.01	0.12; 0.01	–
Lag time	−0.08; 0.05	−0.12; 0.01	−0.11; 0.01
Slope	0.03; 0.48	0.08; 0.07	0.01; 0.02
Maximum absorbance	−0.15; 0.0003	−0.07; 0.12	−0.04; 0.36
CLT	−0.003; 0.94	0.06; 0.17	0.07; 0.15

*In Model 1, the r values are for Spearman correlations; in Model 2, the r values are for Spearman partial correlations adjusting for CRP, LDL-C, age, HbA1c and GGT; in Model 3, the r values are for Spearman correlations adjusting for CRP, LDL-C, age, HbA1c and GGT as well as fibrinogen*.

In Table 3, we present the differences in fibrinogen and clot properties among the 25(OH)D status categories. Fibrinogen γ' tended to increase over the 25(OH)D categories (the first represented those with deficiency, the second those with insufficiency and the third those with sufficient vitamin D) before and after adjusting for possible confounders. Maximum absorbance decreased over increasing 25(OH)D categories, with a significant difference detected between those with vitamin D insufficiency and those with sufficient status before and after adjusting for potential confounders, but not after additional adjustment for total fibrinogen. Neither fasting glucose nor HbA1c differed over the vitamin D status categories (*p* > 0.05).

**Table 3:** 25(OH)D status categories in relation to total and γ' fibrinogen as well as plasma clot properties.

**Model 1**	**Adjusted means with 95% CI determined through GLM**			
	**Deficiency, *n* = 103 (16%)** **<50 nmol/L**	**Insufficiency, *n* = 300 (45%)** **50–75 nmol/L**	**Sufficiency, *n* = 257 (39%)** **>75 nmol/L**	***p*** **value**
Fibrinogen (g/L)	4.34 (3.88–4.81)	4.28 (4.01–4.54)	3.96 (3.66–4.25)	0.21
Fibrinogen γ' (%)	10.7 (8.71–12.7)	11.9 (10.7–13.0)	13.4 (12.2–14.7)	0.05
Lag time (min)	6.90 (6.51–7.30)	6.52 (6.30–6.75)	6.57 (6.33–6.82)	0.24
Slope (AU/s)	9.59 (8.75–10.4)	9.87 (9.39–10.36)	9.83 (9.31–10.4)	0.84
Maximum absorbance (nm)	0.46 (0.43–0.49)	0.47 (0.45–0.48)*	0.43 (0.41–0.45)*	0.02
CLT (min)	57.3 (55.2–59.4)	59.9 (58.7–61.1)	59.5 (58.2–61.0)	0.11
**Model 2**				
Fibrinogen (g/L)	4.18 (3.76–4.61)	4.33 (4.09–4.57)	4.06 (3.80–4.33)	0.35
Fibrinogen γ' (%)	10.7 (8.74–12.7)	11.9 (10.73–13.0)	13.4 (12.2–14.7)	0.05
Lag time (min)	6.93 (6.53–7.32)	6.51 (6.29–6.74)	6.56 (6.32–6.80)	0.19
Slope (AU/s)	9.46 (8.63–10.3)	9.93 (9.44–10.4)	9.91 (9.39–10.4)	0.62
Maximum absorbance (nm)	0.46 (0.43–0.48)	0.47 (0.45–0.48)*	0.43 (0.41–0.45)*	0.02
CLT (min)	57.2 (55.1–59.3)	59.9 (58.7–61.1)	59.5 (58.2–60.8)	0.08
**Model 3**				
Lag time (min)	6.87 (6.47–7.26)	6.46 (6.24–6.69)	6.47 (6.22–6.72)	0.19
Slope (AU/s)	9.55 (8.74–10.4)	9.80 (9.32–10.3)	10.0 (9.50–10.5)	0.63
Maximum absorbance (nm)	0.45 (0.42–0.48)	0.46 (0.44–0.48)	0.43 (0.41–0.45)	0.07
CLT (min)	57.4 (55.3–59.6)	60.1 (58.8–61.3)	59.5 (58.1–60.9)	0.12

*Model 1 for 25(OH)D is adjusted for age, LDL-C, BMI, HbA1c and GGT; Model 2 is adjusted for age, LDL-C, BMI, HbA1c, GGT and CRP; and Model 3 is adjusted additionally for fibrinogen*.

*AU, absorbance units; 25(OH)D, 25 hydroxyvitamin D; CLT, clot lysis time; CI, confidence interval; GLM, general linear model. Conversion: 1 nmol/L 25(OH)D = 0.4 ng/mL 25(OH)D = 0.0004 mg/L 25(OH)D*.

The post hoc test to determine which groups differed was Tukey's unequal N with the ^*^ indicating the groups that differed significantly from each other.

### Gene−25(OH)D Interactions in Relation to Fibrinogen and Plasma Clot Properties

Details of the individual fibrinogen (*FGA, FGB and FGG*) and *FXIII* genes SNPs and their associations with total and %γ' fibrinogen concentration and clot properties have been described elsewhere (39). Here, we focus on the conjoined associations between 25(OH)D and genetics of fibrinogen and *FXIII* with total fibrinogen, %γ' fibrinogen and clot properties. In Table 4, we report only interactions at *p* < 0.05 in models wherein we controlled for other variables.

**Table 4:** *FGB* and *FXIII* gene polymorphisms' interactions with 25(OH)D in relation to plasma fibrin clot properties.

**Interaction**	**Interaction *p* value**	**Genotype**	**Model 1**	**Model 2**	**Model 3**
			**Spearman rho;** ***p*** **value**		
**25(OH)D interaction in relation to total fibrinogen**					
*FGB:* rs1800787	0.04	CC	0.14; <0.0001	−0.1; 0.05	−0.1: 0.05
		CT/TT	0.34; <0.0001	0.04; 0.78	0.04; 0.78
**25(OH)D interaction in relation to lag time**					
*FXIII*: rs5985	0.03; 0.27	GG	−0.013; 0.67	−0.1; 0.06	−0.1; 0.06
		GT/TT	0.12; 0.02	−0.24; 0.01	−0.23; 0.02
**25(OH)D interaction in relation to maximum absorbance**					
*FGB:* rs2227388	0.02; 0.005	AA	−0.25; 0.11	−0.05; 0.91	−0.2; 0.66
		AG/GG	−0.12; <0.0001	−0.08; 0.08	−0.05; 0.29
**25(OH)D interaction in relation to CLT**					
*FGB:* rs2227385	0.007; 0.002	AA	−0.008; 0.84	0.05; 0.3	0.06; 0.22
		AG	−0.15; 0.49	0.13; 0.69	−0.01; 0.79

*p values are, first, for a GLM interaction adjusted for age, LDL-C, HbA1c, GGT, and BMI; and the second, with additional adjustment for fibrinogen. Model 1 represents Spearman correlations; Model 2 shows Spearman partial correlations with adjustment for age, LDL-C, HbA1c, GGT, BMI, and CRP; and Model 3, additionally for fibrinogen*.

*A, adenine; C, cytosine; FGB, fibrinogen beta chain; FXIII, Factor XIII; G, guanine; rs, reference sequence; T, thymine*.

In those carrying the *FGB* wildtype allele at rs1800787, a stronger correlation was detected between 25(OH)D and total fibrinogen than those harboring the variant allele, resulting in an interaction. 25(OH)D did not correlate with lag time in those with homozygote carrier status for the wildtype allele. In contrast, when harboring the variant allele at rs5985 on the *FXIII* gene, a positive association was detected that resulted in an interaction. However, after additional adjustment for fibrinogen, the gene-diet interaction disappeared. An *FGB* gene (rs2227388)–vitamin D interaction was observed concerning maximum absorbance that became stronger after additional adjustment for fibrinogen. In women homozygous for the wildtype allele at rs2227388, no association between 25(OH)D and maximum absorbance was evident. Contrastingly, in those with one or two variant alleles, an inverse correlation was detected. 25(OH)D interacted with *FGA*: rs2227385 in relation to CLT before and after additional adjustment for fibrinogen. However, none of the interactions reached the significance required for adjusting for a multiple testing false discovery rate.

## Discussion

The role of vitamin D in reducing CVD risk has been recognized (8). In particular, among the favorable pleiotropic effects of vitamin D on CVD, researchers have explored the relationship between vitamin D and total fibrinogen concentrations in small patient populations with large scale epidemiological studies remaining relatively limited. In this study, we determined the relationships between total fibrinogen and—to our knowledge for the first time—the γ' splice variant of fibrinogen, and clot properties and 25(OH)D concentrations in generally healthy, Black South African women, among whom vitamin D deficiency was common. We observed an inverse, albeit weak, relationship between 25(OH)D and fibrinogen as well as clot density as indicated by maximum absorbance and a positive association between 25(OH)D and γ' fibrinogen. This was supported by observing differences in maximum absorbance and γ' fibrinogen among vitamin D status groups as well. Partial correlations with adjustment for, among others, CRP, an inflammatory marker, and HbA1c, which is a long-term indicator of glucose control, indicated that vitamin D only correlated with γ' fibrinogen and lag time. Even though several correlations were statistically significant, possibly due to a loss in statistical power, less differences among fibrinogen and clot properties were observed when the study population was subdivided according to their vitamin D status. Several gene-diet interactions were detected at the *p* < 0.05 level, but did not stand after correction for false discovery rate.

Here we also report an inverse association between 25(OH)D and fibrinogen before adjusting for other variables. Our study and the results obtained are in line with those of Parikh et al. (25) obtained in 701 Black and White adolescents living in the southeastern region of America; of Mellenthin et al. (19), who investigated 2,723 men and women from a general European population; of Lo Gullo et al. (26), who sampled 27 rheumatoid arthritis patients and 41 healthy controls; of Blondon et al. (12) who studied 6,554 multiethnic American adults free of CVD; and of de Oliveira et al. (20), who investigated 5,870 English adults. Mellenthin et al. (19), Blondon et al. (12) and de Oliveira et al. (20) are, to our knowledge, the only investigations similar to ours, that are, of observational nature involving adult participants from general populations. The observational study of Mellenthin et al. (17) did not include models adjusting for co-variates. Oliveira (18) reported that the relationship between fibrinogen and vitamin D remained whereas Blondon et al. (12) found that it did not after adjusting for age and other demographic variables. Evidence for a relationship between vitamin D and fibrinogen from intervention trials predominantly involved small groups of ill patients, with conflicting results. Even though Akin et al. (21), Fornari et al. (27) and Barnes et al. (44) reported measuring both vitamin D and fibrinogen concentration, they did not report on any association between the variables. An intervention trial conducted in Helsinki, Finland, administered 25(OH)D supplements for 6 months to 218 long-term care patients who were chronically bedridden, but failed to alter fibrinogen concentrations (22). A double-blind, randomized, controlled trial conducted over a 12-week period in 90 type 2 diabetic men and women was used to investigate the effects of vitamin D either with or without extra calcium on fibrinogen (23). Compared to the baseline values, but not among the subgroups, fibrinogen decreased in both those consuming a vitamin D-fortified Persian yogurt drink (or *doogh*, containing 500 IU vitamin D_3_ and 150 mg calcium/250 mL) and calcium plus vitamin D_3_-fortified *doogh* (containing 500 IU vitamin D_3_ and 250 mg calcium/250 mL). The reduction was seen in both groups that also received calcium; future research is therefore needed to tease out the effects of vitamin D supplementation *per se*. Soare et al. (24) failed to detect a difference in fibrinogen between vitamin D-deficient (25(OH)D >20 ng/mL) and -sufficient groups (≥20 ng/mL) in 62 patients with inflammatory bowel disease. Asymptomatic or mildly symptomatic severe acute respiratory syndrome coronavirus 2 RNA positive vitamin D deficient participants were randomized to receive cholecalciferol for a week to correct the deficiency (n = 16) or a placebo (n = 24) (45). Vitamin D supplementation decreased fibrinogen levels, but did not affect other inflammatory markers (45). To summarize, both observational and experimental studies' results allude to an inverse relationship between fibrinogen and vitamin D which is in line with our findings.

Regarding γ' fibrinogen and clot properties, our study is the first to allude to a relationship with vitamin D. Here we expressed γ' as a percentage of total fibrinogen concentrations. In individuals with varying total fibrinogen concentration, the γ' fibrinogen concentration will likely also vary as its concentration is dependent on the total amount of fibrinogen produced. When expressing it as % of total fibrinogen, the potential confounding effect of differing total fibrinogen concentrations is excluded. Previously we reported our observations that while γ' was high when total fibrinogen concentration was high; a decreased γ'/total fibrinogen ratio was observed at the highest total fibrinogen concentrations (32). This finding explains the seemingly conflicting relationships i.e., the positive relationship detected between vitamin D and γ' and the negative relationship between vitamin D and total fibrinogen.

Mechanisms by which vitamin D may influence fibrinogen and clot properties are not known and should be explored in future research to determine the biological relevance of our observations. Possible mechanisms for the inverse association between vitamin D and fibrinogen and clot density might be through inflammation and changes in the glycemic milieu. Thus, regarding inflammation, fibrinogen is itself a downstream inflammatory molecule and therefore directly affected by the suppressor effect of vitamin D on the production of cytokines. *In vitro* evidence demonstrated that the active metabolite of vitamin D, 1, 25(OH)D, and its analogs could couple to the vitamin D receptor and subsequently downregulate several inflammatory cytokines, including tumor necrosis factor-α, and interleukin 1 and 6 (46–49). This theory is partly supported by our results, because after adjustment for the inflammatory marker CRP (which correlated strongly with fibrinogen), the relationship between fibrinogen and vitamin D disappeared. Moreover, vitamin D's effects on insulin and fasting blood glucose could improve plasma clot properties. Heightened insulin sensitivity (16, 17) could change the glycation of the activated fibrinogen molecules, thereby improving clot structure (making it less dense and therefore less resistant to degradation by plasmin) (50, 51). However, we did not detect any difference in HbA1c or fasting glucose among the vitamin D status groups. Furthermore, the associations between fibrinogen and clot properties remained after adjustment for age and HbA1c alone (results not shown), possibly because the participants did not have diabetes. Therefore, the HbA1c concentrations were normal for most in our study population, so adjusting for it had little effect. To explore the latter mechanism in future, one would probably need to sample diabetic patients and not apparently healthy individuals with normal glycation levels. Whatever the mechanism, decreased plasma total and γ' fibrinogen and improved plasma clot properties (i.e., here vitamin D was associated with less dense plasma clot configurations) may have implications for preventing CVD.

Currently, there is no proof of a direct pathway by which 25(OH)D or its metabolized product, 1, 25(OH)D, influences the expression of the fibrinogen genes, *FGA, FGB*, and *FGG*. It seems more plausible that fibrinogen gene expression is influenced by cytokines (36); because they are downregulated by vitamin D (46–49), this might modulate the genes. In terms of *FXIII*'s genetic determinants and possible modulators, apart from the work from our research group, we could not find any other publications. Even though we detected some vitamin D–gene interactions, we question their significance because they did not remain after correction for false discovery rate. Therefore, the value of our findings in terms of nutrigenetics using a candidate gene approach remains to be clarified in studies with more power or through the use of genome wide association studies investigated interactions with vitamin D.

Some limitations that could be addressed by future research must be acknowledged. As women are more likely to develop skeletal disorders associated with low 25(OH)D status than men and the study budget being constrained, we only included women. Our study's power to detect interactions with SNPs was low because we had access to a relatively small subset. Its strengths include that we investigated an ostensibly healthy population of Black South Africans known to present with high fibrinogen and low vitamin D owing to skin pigmentation, thereby increasing our chances of detecting an association should it exist. While total fibrinogen concentrations here reflect functional fibrinogen, the γ' fibrinogen levels are antigen based. Even though the absolute percentage may be slightly different if one measured total fibrinogen with an antigen assay, all samples were analyzed with the same methods, thus allowing comparison between individuals with low or high γ' fibrinogen. We, furthermore, report % γ' fibriniogen for correlations and relationships since the ratio is thought to be more physiologically relevant than the absolute concentration. Currently, our report is the only one of its kind to have considered the γ' fibrinogen molecule and plasma clot properties.

## Conclusions

Low vitamin D (7, 52, 53) and elevated total (54–62) and γ' fibrinogen (63–67) as well as dense blood clots resistant to lysis (68, 69) are independently associated with unfavorable health outcomes, including CVD. The current study expanded on existing knowledge of the putative role of vitamin D in terms of fibrinogen and the functionalities of fibrinogen in terms of fibrin clot properties, many of which have not been studied before with respect to vitamin D. In the present cross-sectional study, we exposed an inverse association between 25(OH)D and fibrinogen concentrations as well as clot density. Moreover, γ' fibrinogen correlated positively with 25(OH)D. Because the association between fibrinogen and clot properties failed to remain statistically significant after adjustment with variables including CRP, we believe that plausible mechanisms that could be explored in future are through the anti-inflammatory properties of vitamin D. γ' fibrinogen increased whereas clot density decreased over the 25(OH)D categories before and after adjustment for confounders. Because the observed relationships were weak, the clinical significance of the findings should be determined in future epidemiological and intervention trials. The results reported here could inform future intervention trials in which responsible exposure to sunlight, the inclusion of vitamin D-rich foods and/or whether supplementation or fortification with vitamin D analogs are explored in relation to blood coagulation might ultimately offer therapeutic options for those with a high risk of developing heart disease. The observed relationship between vitamin D and CVD could ultimately reveal promising mechanisms—that might include a link to fibrinogen and plasma clot configurations—which could be modified by lifestyle or supplementation and fortification.

## Data Availability Statement

The data analyzed in this study is subject to the following licenses/restrictions: Raw data were generated at the North-West University. Obtained data supporting the findings of this study are available from the PI of the study after obtaining the necessary ethical approval. Due to Ethical restrictions and the POPI act data are not publicly available. Requests to access these datasets should be directed to lanthe.kruger@nwu.ac.za.

## Ethics Statement

The studies involving human participants were reviewed and approved by Health Research Ethics Committee of the Faculty of Health Sciences, North-West University. The patients/participants provided their written informed consent to participate in this study.

## Author Contributions

Conceptualization: CN-R. Methodology: PR, CN-R, ZL-L, IK, and MP. Statistical analysis and writing—original draft preparation: PR and CN-R. Resources and funding acquisition: IK and MP. Data curation and project administration: IK. Writing—review and editing: ZL-L, IK, and MP. All authors contributed to the article and approved the submitted version.

## Funding

This research was funded by SANPAD [South Africa–The Netherlands Research Program on Alternatives in Development (08/15)], the South African National Research Foundation (NRF GUN No. 2069139 and FA2006040700010), self-initiated research grants from the South African Medical Research Council, North-West University, Potchefstroom, South Africa, the Population Health Research Institute, Ontario, Canada and the Academy of Medical Sciences UK (Newton Fund Advanced Fellowship Grant [AMS-NAF1-Pieters to MP].

## Conflict of Interest

The authors declare that the research was conducted in the absence of any commercial or financial relationships that could be construed as a potential conflict of interest.

## Publisher's Note

All claims expressed in this article are solely those of the authors and do not necessarily represent those of their affiliated organizations, or those of the publisher, the editors and the reviewers. Any product that may be evaluated in this article, or claim that may be made by its manufacturer, is not guaranteed or endorsed by the publisher.
